# Epigenetic Regulatory Dynamics in Models of Methamphetamine-Use Disorder

**DOI:** 10.3390/genes12101614

**Published:** 2021-10-14

**Authors:** Subramaniam Jayanthi, Michael T. McCoy, Jean Lud Cadet

**Affiliations:** Molecular Neuropsychiatry Research Branch, NIDA Intramural Research Program, Baltimore, MD 21224, USA; mmccoy@intra.nida.nih.gov

**Keywords:** addiction, DNA methylation, epigenetics, HDAC inhibitors, histone acetylation, histone methylation, methamphetamine

## Abstract

Methamphetamine (METH)-use disorder (MUD) is a very serious, potentially lethal, biopsychosocial disease. Exposure to METH causes long-term changes to brain regions involved in reward processing and motivation, leading vulnerable individuals to engage in pathological drug-seeking and drug-taking behavior that can remain a lifelong struggle. It is crucial to elucidate underlying mechanisms by which exposure to METH leads to molecular neuroadaptive changes at transcriptional and translational levels. Changes in gene expression are controlled by post-translational modifications via chromatin remodeling. This review article focuses on the brain-region specific combinatorial or distinct epigenetic modifications that lead to METH-induced changes in gene expression.

## 1. General Introduction

Methamphetamine (METH) is a powerful psychostimulant that belongs to the class of amphetamine-type stimulants (ATS) and has a high potential for abuse. The other ATS include amphetamine and 3,4-methylenedioxymethamphetamine (MDMA, ecstasy). At low therapeutic doses, METH can cause elevated mood and increased alertness, improved concentration, and increased energy in fatigued individuals [1]. In the past few years, the prevalence of METH abuse has exceeded that of cocaine use worldwide because it produces euphoria that lasts longer due to its 12-h half-life compared to the 90 min half-life of cocaine [2]. METH is also popular because of cost differences [3,4] and the ease of its synthesis from precursors such as ephedrine (derived from the plant Ephedra sinica), pseudoephedrine, or 1-phenyl-2-propanone [5]. Recently, new alternative “designer precursors”, including methyl α-phenylacetoacetate (also known as MAPA) [6] and its optical isomers, have been used by clandestine laboratory operators to circumvent federal controls placed on other known precursors. With the development of various synthetic production methods, METH continues to dominate global market scenes. According to reported seizure data for 2015–2019, METH accounted for 72% of all global ATS-related seizures. The three countries with most ATS-related seizures are the United States (US), Thailand, and Mexico [7].

### 1.1. Epidemiology of METH Use

Prevalence of METH use is often used to gauge the significance of its abuse. According to the United Nations Office on Drug and Crime (UNODC, 2021), 27 million people worldwide used amphetamines, suggesting a significant increase in the geographical spread of METH trafficking at the global level [7]. METH use in the USA displays a stable trend, with the highest incidence of METH use among the general population aged between 15 and 64 [8,9]. Of note, it has reported that two-thirds of METH users progress to develop a METH-use disorder (MUD) [8]. Public health surveillance systems have documented significant increases in METH-related emergency department (ED) visits compared to marijuana, alcohol, heroin, and cocaine [9]. The number of treatment admissions for MUD has also increased [10]. The overall number of deaths attributed to the use of psychostimulants (primarily METH and excluding opioids) in the USA increased by six-fold in 2019 compared to 2010 [7].

### 1.2. Clinical Presentations of METH-Use Disorder

The clinical presentations of MUD depend on the route of administration, (smoked, snorted, swallowed, or injected), patterns of drug use, and amounts of drug taken. When smoked, snorted, or injected, the user experiences, within minutes, an intense “rush” or “flash” that is described as extremely pleasurable. With oral intake, these effects are less prominent and occur after 2–4 h. Administration of METH can also result in cardiovascular, cerebrovascular, and other autonomic dysfunctions [11].

METH use usually starts as a recreational activity wherein users take relatively low doses at relatively long intervals between drug-taking behaviors. Users progress over time to taking the drug in binges followed by intervals of abstinence after they run out of drug. Binges can also be accompanied by crashes during which individual users can feel various adverse consequences that include mood disturbances and intense craving. These withdrawal symptoms often serve to accelerate the escalation of METH taking that is accompanied by significantly shortened intervals between successive METH injections. METH doses self-administered during recreational use in humans are estimated at 20–40 mg, which, for a 60–80 kg person, are equivalent to 0.25–0.67 mg/kg doses [12]. Hart et al. (2001) [13] have reported that METH can cause positive drug effects in subjects even at a very low dose equivalent to 0.06–0.08 mg/kg [13]. A binge dose can be as high as 0.5–1 g/day and METH self-administration might occur 4–6 times per day over a 24-h period [3,4].

During withdrawal, most people experience depression, anxiety, tiredness, and intense craving for the drug [14]. The clinical scenario can also include complex cardiovascular and cerebrovascular symptoms [11] in addition to the psychostimulatory effects of the drug. Pre-existing psychiatric comorbidities are also known phenomena of the MUD diathesis [15,16].

As mentioned earlier, only some people who use METH develop MUD [13]. The development of MUD is thought to be related to genetic and environmental factors that result in either resilient or susceptible phenotypes during the chronic use of rewarding drugs [17]. The switch from recreational use to meeting clinical criteria for MUD is likely related to epigenetically driven transcriptional and translational changes that can impact synaptic functions and responses to METH over decades. Recently, we published a brief review on the epigenetic landscape of MUD [18] where we mainly focused on animal models of METH administration. In this review, we focus on both pre-clinical and clinical aspects of METH-induced chromatin modifications. Herein, we have provided individual tables that summarize the experimental design, assays, and results of all the epigenetic alterations (histone, DNA and non-coding RNA modifications) in MUD. Besides, this review addresses the gender differences and the effects of pre-natal exposure of METH in relation to chromatin modification. Overall, we discuss potential METH-induced epigenetic alterations that might serve as substrates to promote a permissive state that can lead to the behavioral manifestations called addiction.

## 2. Alterations of Chromatin Dynamics in METH-Use Disorder

### 2.1. Brief Introduction

The term “epigenetics” was coined by Conrad Waddington in 1942 to explain “the causal interactions between genes and their products”, which he defined as changes in phenotype without changes in genotype. A reprint of this original article was published in 2012 [19]. Epigenetic regulation refers to stable and heritable transcriptional changes in gene expression that are not secondary to alterations in DNA sequences. Epigenetic changes enable the same DNA sequences to function differently under different circumstances and promote changes in behaviors, learning and memory formation, and in cognitive functions.

**Figure 1:**
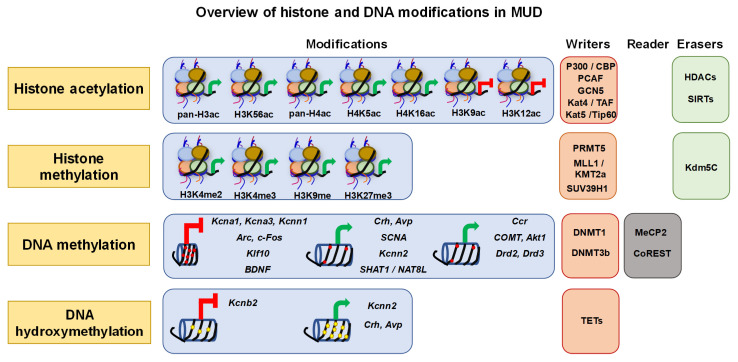
Illustration of aberrant alterations in METH-induced epigenetic modifications and effectors represented by “writers”, “readers”, and “erasers”. Epigenetic writers include histone acetyl-transferases (HATs: p300/CBP; PCAF; GCN5; TAF/Kat4; Tip60/Kat5), histone methyl-transferases (HMTs: PRMT5; MLL1/KMT2a; SUV39H1), DNA methyltransferases (DNMT: DNMT1; DNMT3b), ten-eleven translocase (TET: TET1, 2 and 3), readers include, corepressor for the RE1-silencing transcription factor (CoREST), methyl-binding protein (MBP: MeCP2) and erasers include, histone demethylase (HDM: Kdm5c), histone deacetylases (HDACs). The green arrow represents transcriptional activation and red block-head arrow represents transcriptional repression. The red circle on DNA strand (DNA methylation) indicate methyl-cytosine; yellow pentagon on the DNA strand (DNA hydroxymethylation) indicates hydroxymethyl-cytosine. ac: acetylation; me: methylation.

The chromatin can undergo a complex network of events that are responsible for registering and maintaining gene regulation via “marks”. As illustrated in Figure 1, the specific editing process is carried out by “writers” and “erasers” that modify the histone proteins that are tightly bound to the DNA [20,21]. Writers include histone acetyl transferase (HATs), histone methyl transferase (KMTs/HMTs), and SET family of methyl transferase (SUV). These enzymes target promoters and enhancers to facilitate transcriptional activation [22,23,24]. Erasers include histone deacetylases (HDACs) and demethylases (KDM), which govern transcriptional repression [25,26]. In addition, a set of proteins, chromo and bromodomains, are called “readers” and are located within the chromatin. They function as chromatin remodelers and adaptor proteins by interacting with transcriptional machinery (reviewed by Zhu et al. 2020 [27]).

In the following pages, we will discuss the identified core molecular actors that appear to significantly contribute to the development of MUD (see Figure 1). These include, modifications to the histone proteins along with changes in DNA (methylation and hydroxymethylation). These alterations influence the overall chromatin dynamics to make DNA more (euchromatin) or less (heterochromatin) accessible to the transcriptional machinery, with consequent activation or suppression of transcription [28]. These changes can significantly impact the development and/or progression of diseased states [29].

### 2.2. Histone Modifications in METH-Use Disorder

Histone modifications are covalent modifications where the N-terminal tail of histone proteins undergoes post-translational changes by addition of acetyl, methyl, ubiquitin, phosphoryl, sumoyl, ADP ribosyl moieties along with deamination and proline isomerization [30,31,32].

#### 2.2.1. Histone Acetylation

Histone modifications are poised to play a role in setting up the dynamic responsivity and range of brain regulation of physiology and behavior. The first histone modification was identified in the mid-1960s by Allfrey et al. (1964) who mulled over the role of acetylated histones in transcription regulation [33]. Acetylation of histone proteins requires a balance between the activities of both HATs and HDACs [34]. Histone acetylation is generally associated with increased gene expression while a hypo-acetylated state results in decreased gene expression [35,36,37]. Under normal conditions, HAT and HDAC are maintained in a balanced homeostatic state in the neuron. However, in the neurodegenerative state the acetylation homeostasis is disturbed [38,39]. HDACs are critical regulators of gene expression and play important roles in the pathophysiology of several neurodegenerative diseases. These include Parkinson’s [40], Alzheimer’s [41], dementia [42], schizophrenia [43], bipolar disorder [44], and depression [45]. Similarly, the most investigated chromatin modification in drugs of abuse is histone acetylation [46,47,48,49,50]. Histone acetylation states have been investigated after various METH dosing regimens and in the presence of HDAC inhibitors (HDACi).

##### Acute Effects of METH on Histone Acetylation

Scientists in the Cadet laboratory have published several papers in which they interrogated the status of histone modifications using experimenter-administered METH in rodent models [51,52,53,54]. Most of these reports pertain to deacetylated histones, which lead to a reduction in transcription activity [36,37].

In 2019, González and colleagues reported that an acute injection of METH (1 mg/kg) in mice increased HDAC1, HDAC2, and pan-acetylated histone H3 protein levels in the prefrontal cortex (pCTX) whereas pan-acetylated histone H4 protein showed a decrease [51] (see Table 1). However, in the nucleus accumbens (NAc), a large single dose of METH (20 mg/kg) in rats decreased HDAC1 but increased HDAC2 protein levels [53]. These results were associated with decreased abundance of histone H3 acetylated at lysine 9 (H4K5ac) and at lysine 18 (H3K18ac). In contrast, there was increased abundance of histone H4 acetylated at lysine 5 (H4K5ac) and at lysine 8 (H4K8ac) [53]. These observations suggest that METH can cause differential changes in different histone acetylation on histone proteins and support the idea that changes in chromatin dynamics can occur in independent histone-specific fashion [51,53,55,56,57]. These findings also suggest that METH can impact gene expression according to its impact on various histone markers at specific gene promoters. This suggestion is supported by chromatin immunoprecipitation (ChIP) studies after METH administration. Specifically, pull-down assays using a pan-H3ac antibody revealed a significant enrichment of this marker at the promoters of Drd2, Adra1a, Hcrtr1, and Hrh1 genes but decreased abundance at the Hrh3 promoter [51]. Moreover, ChIP using a pan-H4ac antibody documented increased enrichment at Drd1 and Hcrtr1 promoters but decreased abundance at Hcrtr2, Grin1, and Hrh3 promoters [51]. These results underlie the potential complex epigenetic responses when METH is administered.

It is also interesting to compare the epigenetic effects of METH to those of modafinil, a drug used clinically to treat ADHD [58], narcolepsy [59]. Modafinil has also been used in clinical trials for treatment of cocaine [60,61] and METH dependence [62]. Modafinil treatment did not alter the protein levels of pan-H3Ac, H4ac, or of HDACs-1 and 2. ChIP data from modafinil-treated mice using pan-H3ac and pan-H4ac identified similar results to those reported for METH-treated animals, with additional findings of increased enrichment of H3ac at Drd1 and Adra1b promoters and of H4ac at the Drd2 promoter. The authors also reported decreased H4ac at the Hrh1 promoter after modafinil treatment. Drug-induced comparable and differential epigenetic changes might be responsible, in part, for some of the behavioral similarities and differences reported after administration of various stimulants [51].

In vitro studies have also been used to study the epigenetic effects of METH. Exposure to METH (10uM) upregulated HDAC1, HDAC4, and HDAC6 expression, but decreased HDAC5 expression levels in astrocytes [63]. The paper also reported increased p300, a histone acetyltransferase, but decreased expression of the HATs, PCAF, and GCN5 [63]. Taken together, these studies indicate that METH can have differential effects on epigenetic markers both in vivo and in vitro, supporting the idea that epigenetic mechanisms are very important events to understand when planning future therapeutic interventions against MUD.

##### Chronic Effects of METH on Histone Acetylation

In addition to investigating the effects of the administration of single doses of METH, González et al. (2018) [64] also studied alterations in histone acetylation after repeated injections of METH over several days. They focused their analysis on various receptors involved in cognitive control such as the dopamine (Drd1 and Drd2), adrenaline (Adra1a and Adra1b), orexin (Hcrtr1 and Hcrtr2), histamine (Hrh1 and Hrh3), and glutamate (Gria1 and Grin1) receptors in the medial pCTX. They found that repeated injections of METH (1 mg/kg, daily for 7 d) followed by withdrawal for 4 days significantly impacted recognition memory. However, modafinil, an FDA-approved drug used for treatment of narcolepsy did not elicit similar behavioral changes. They showed further that METH caused increased H4ac enrichment at Drd1, Hcrtr1, and Grin1 promoters but less enrichment of H3ac on the promoters of Drd2, Hcrtr1, Hcrtr2, Hrh1, Hrh3, and Grin1 in the medial pCTX [64]. However, they found no differences in the effects of the two drugs on the protein expression of pan- H3ac and H4ac [64]. These results support the notion that enrichment at gene promoters is not directly related to the levels of the histone protein under investigation. A recent ChIP-chip analysis using a rat model of METH-induced behavioral sensitization discovered increased H3 acetylation in a large number of gene promoters (241 genes) and increased H4 acetylation in a fewer number of gene promoters (10 genes) in the pCTX [65]. Interestingly, H4 hyperacetylation was found to contribute to increased expression of Angp32a, a gene is involved in the regulation of synaptic plasticity and memory [65].

Additional studies to test the effects of METH on specific acetyl marks on lysine residues located on histone tails have revealed that acute and repeated METH exposure can cause enrichment of H4K5ac on the promoter regions of several immediate early genes (IEGs), with some differences in the observed patterns for the two conditions [47].

Interestingly, repeated METH injections caused decreased H4K5ac protein levels and of its binding to the promoter regions of AMPA and NMDA glutamate receptor subunits [66].

Chronic administration of METH mimicking the “binge” model showed reduction in the mRNA levels of Hdac8 (Class I), Hdac9 (Class IIa), Hdac6, and Hdac10 (Class IIb), Sirt2, Sirt5 and Sirt6 (Class III), and Hdac11 (Class IV) in the dorsal striatum (DStr), a region for habit formation [67].

The self-administration (SA) animal model has been used to model drug addiction in humans because animals escalate their drug intake, spend considerable time to obtain the drug, and will continue to take drugs despite the adverse consequences [68]. Using the SA approach, Cadet and his group have shown that METH can cause changes in histone deacetylation in brain regions that participate in the reward circuitry [69]. This approach involved allowing rats to self-administer and escalate METH intake followed by contingent footshocks as representative of adverse consequences and a subsequent period of METH-seeking behavior after 30 days of forced withdrawal from METH SA. The investigators reported that there were increased levels of HDACs (Hdac2, Hdac8, and Hdac9) in the dorsal striatum but decreased levels of HDACs (Hdac1, Hdac5, Hdac7, Hdac10, and Hdac11) in the nucleus accumbens of compulsive METH takers [69]. These observations support the notion that HDACs are important participants in mediating certain aspects of METH-induced behavioral consequences associated with METH-taking behaviors.

##### Role of HDAC Inhibitors in METH Exposure

Given the critical role of HDACs in SUDs, it will be important to investigate how manipulations of HDAC activities might influence the drug-taking behaviors in animal models of addiction. If successful, these studies can be followed in experiments using clinical settings. In fact, since there are several HDAC inhibitors (HDACis) that are in clinical use against cancer, it should be possible to use the drugs with the least side-effects in present clinical practice.

METH use is known to be accompanied with behavioral sensitization that can last for several weeks to months in rodents [65,70]. Interestingly, the class I HDACi, valproic acid (VPA), can attenuate behavioral sensitization responses [71]. In another study, it was reported that microinjections of NaB and VPA into the prefrontal cortex, ventricle, amygdala, and dorsal striatum but not into the hippocampus could attenuate METH-induced hyperactivity [44]. Together, these results further support a role for HDAC in the acute and chronic behavioral effects of the drug.

As previously reported, chronic METH injection resulted in decreased H4K16ac binding and expression of GluA1 and GluA2 and using VPA-normalized binding and expression [66]. A specific HDAC6i that is thiazolidinedione-based has been shown to normalize the abundance of acetylated α-tubulin and to reverse METH-induced morphological changes in a neuroblastoma cell line [72]. Moreover, Kim et al. (2020) [73] used another HDAC6i, MeBib derived from a benzimidazole scaffold, and were able to reduce the METH SA by rats [73].

Many investigators have sought to determine the role of HDACs in the effects of METH by using HDAC knockout mice. For example, using a HDAC2 knockout mouse model, Torres et al. (2016) [54] investigated the effects of METH on HDACs and found that METH decreased the expression of Hdac3 and Hdac8 (class I), Hdac4 and Hdac7 (class IIa), as well as and Hdac11 (class IV) in wild-type mice. In contrast, those changes were normalized in HDAC2 KO mice [54].

Other investigators have used METH SA in rats to assess the role of HDACs in drug-taking behaviors [74]. They found that Hdac5 overexpression in the dorsal striatum using viral vectors increased whereas Hdac5 knockdown decreased METH-seeking behavior [74]. In addition, HDAC5 knockdown rats exhibited increased HDAC1, HDAC4, and HDAC5 target genes, namely Gnb4 and Suv39h1 [74].

##### Role of HATs in METH Exposure

Many investigators have examined the potential involvement of HATs in METH-mediated gene transcription. For example, Shibasaki et al. (2011) [75] reported that METH increased histone H3 acetylation and upregulated the expression of several synaptic plasticity genes known to be regulated after acute or repeated injections of METH [76,77,78]. Cadet and co-workers have also provided evidence that METH SA is associated with up-regulation of the HATs, Kat4, and Kat5 in the DStr [69]. Recently, an in vitro study by Doke et al. (2021) [63] using human primary astrocyte reported that METH significantly upregulated p300/CBP and down-regulated GCN5 and PCAF levels. The authors also reported that METH exposure upregulated global acetylation levels of histone H3 lysine, H3K27ac, H3K56ac and down-regulated H3K14ac. These observations support the notion that METH can cause a combination of histone modifications that can control chromatin dynamics during acute or repeated injections of METH.

One of the most extensively studied signaling pathway after METH administration involves cAMP activation, CREB (cAMP response element binding protein), and CBP (CREB binding protein) [52,79,80]. p300/CBP serves as an adaptor between CREB and the transcription initiation complex [81]. METH administration was found to increase CREB phosphorylation in both ventral and dorsal striata [52,79]. In addition, these investigators observed increased recruitment of pCREB onto the promoter regions of c-fos, fosB, Bdnf and Syp [79], and Cartpt [52].

#### 2.2.2. Histone Methylation

Cross-talks between different types of histone modifications are often observed in various brain regions that control the sensitivity to the psychostimulant exposure, (reviewed by Hitchcock and Lattal, 2014 [82]). Ikegami et al. (2010) [83] found that intermittent subcutaneous injections of METH-induced behavioral sensitization in mice and significantly increased the mRNA expression of the chemokine receptor CCR2, implicated in drug-rewarding properties [84,85,86]. Activation of CCR2 was due to increased trimethylation of histone H3 at lysine 4 (H3K4me3) at the promoter site of CCR2 [83]. Consistent with this result, METH-associated memory was accompanied by increased H3K4me2/3 in the NAc [87]. These changes were secondary to augmented expression of the “writer” enzyme, histone methyltransferase myeloid/lymphoid, or mixed-lineage leukemia 1 (MLL1), but decreased expression of “eraser” enzyme, histone demethylase KDM5C. These two enzymes are known to regulate H3K4 methylation [88]. To validate the role of H3K4 methylation modifiers, the investigators used siRNA-mediated focal, intra NAc knockdown of Mll1 that led to reduced H3K4me3 and reduced cfos and Oxtr mRNA levels; however knockdown of Kdm5C resulted in hypermethylation of H3K4 [87]. Together, these findings identified histone methylation as a novel molecular mechanism that can also influence METH-induced behavioral sensitivity.

It should be pointed out that, in the DStr, METH SA caused increased H3K4me3 protein but no changes in H3K4me3 binding on the promoter of cfos [79]. These discrepant findings suggest that there are regional effects of METH on the epigenetic regulation of gene expression in the brain. The data suggest that experimenter-injected drug and drug self-administration might result in different epigenetic consequences and associated changes in gene expression.

**Table 1:** Histones modifications affected by METH exposure.

Source	Experimental Design	Comparison	Assay and Results				Ref.
			**WB**	**H4K5ac Enrichment ChIP-PCR**	**H4K5ac Enrichment ChIP-Seq**	**RT-PCR**	
Rat male DStr	SM-Saline for 3 wk followed by METH (5 mg/kg), i.p.	↑ SM vs. CT	ARC and c-FOS	*Arc, Crem, Egr2, c-Fos, Npas4, and Nr4a3*	*Crem, DNAjb5, Egr2, c-Fos, Npas4, Nr4a3, and Nts*	*Arc, Crem, Egr2, c-Fos, Npas4, and Nr4a3*	[47]
	MS-ETH escalated doses (0.5 to 3 mg/kg) for 3 wk followed by saline, i.p.	↑ MS vs. CT	ARC and c-FOS				
		↓ MS vs. CT		*Egr2*			
		↑ SM vs. MS		*Arc, Crem, Egr2, c-Fos, Npas4, and Nr4a3*		*Arc, Crem, Egr2, c-Fos, Npas4, and Nr4a3*	
	MM-METH escalated doses (0.5 to 3 mg/kg) for 3 wk followed by challenge METH (5 mg/kg), i.p.	↑ MM vs. CT	ARC and c-FOS	*Egr2, Npas4, Nr4a3*		*Arc, Egr2, c-Fos, Npas4, Npb, Nr4a3, and Nts*	
		↑ MM vs. MS		*Crem, Egr2, c-Fos, Npas4, and Nr4a3*		*Arc, Egr2, c-Fos, Npas4, and Nr4a3*	
		↑ MM vs. SM			*Nr4a3*	*Nr4a3*	
		↓ MM vs. SM				*Arc, Crem, Egr2, and c-Fos*	
			**WB**	**Pan-H3ac Enrichment ChIP-PCR**	**Pan-H4ac Enrichment ChIP-PCR**	**RT-PCR**	
Mouse pCTX	Single dose METH (1 mg/kg, s.c.) for 1 h, co-treatment with modafinil (Mod) (90 mg/kg i.p.)	↑ METH vs. CT	pan-H3ac, HDAC1, HDAC2, and GLUN1	*Drd2, Adra1a, Hcrtr1, and Hrh1*	*Drd1 and Hcrtr1*	*Drd1, Adra1a, Hcrtr1, Hrh1, Hdac1, and Hdac2*	[51]
		↓ METH vs. CT	pan-H4ac	*Hrh3*	*Hcrtr2, Grin1, and Hrh3*		
		↑ METH + Mod vs. CT	pan-H3ac, HDAC1, HDAC2, and GLUN1	*Drd1, Drd2, Adra1b, Hcrtr1, and Hrh1*	*Drd2 and Hcrtr1*	*Drd1, Adra1a, Hcrtr1, Hrh1, Hdac1, and Hdac2*	
		↓ Mod vs. CT	H4ac	*Hrh3*	*Hcrtr2, Grin1, Hrh1, and Hrh3*	*Hdac2*	
			**WB**	**pCREB ChIP-PCR**			
Rat male NAc	Single dose of METH (10 mg/kg) for 1, 2, 3, and 4 wk, i.p.	↑ METH vs. CT	pCREB	*Cartpt (4 wk)*			[52]
			**WB**				
Rat male DStr	Single injection METH (20 mg/kg) for 1, 2, 4, 8, 16, and 24 h, i.p.	↑ METH vs. CT	1, 2, 4, 8, 16, and 24 h—H4K5ac 4, 8, 16, and 24 h—HDAC2				[53]
		↓ METH vs. CT	1, 2, 4, 8, 16, and 24 h—H3K9ac 8, 16, and 24 h-H3K18ac 1, 2, 4, 8, and 16 h—HDAC1				
			**RT-PCR**				
Mouse male NAc	Single dose of METH (20 mg/kg) for 1, 2, and 8 h in HDAC2 KO and WT, i.p.	↓ WT vs. CT	1 h—*Hdac3, Hdac4, Hdac7, and Hdac11* 2 h—*Hdac4, Hdac7, and Hdac11* 8 h—*Hdac3, Hdac4, Hdac8, and Hdac11*				[54]
		↑ WT vs. CT	1, 2, and 8 h—*Hdac6*				
		↓ KO vs. CT	1 h—*Hdac4*				
		↑ KO vs. CT	1 h—*Hdac7 and Hdac11* 2 h—*Hdac8 and Hdac11* 8 h—*Hdac3, Hdac4, Hdac6, and Hdac11*				
		↑ WT vs. KO	1 and 2 h—*Hdac6*				
		↑ KO vs. WT	1 h—*Hdac3 and Hdac7* 2 h—*Hdac3, Hdac4 and Hdac8* 8 h—*Hdac3, Hdac4, and Hdac7*				
			**WB**	**RT-PCR**			
Human primary astrocytes (cerebral cortex)	Single dose of METH (10 µM), co-treatment with Piracetam (10 µM),	↑ METH vs. CT	HDAC1, HDAC4, HDAC6, p300/CBP, pan-H3ac, and H3K56ac	*Hdac1*			[63]
		↑ METH + Piracetam vs. CT	HDAC2, HDAC3, HDAC4, pan-H3ac, and H3K56ac				
		↓ METH vs. CT	HDAC5, PCAF, GCN5 and H3K14ac				
		↓ METH + Piracetam vs. CT	HDAC5, PCAF, and H3K14ac				
			**WB**	**pan-H3ac enrichment ChIP-PCR**	**pan-H4ac enrichment ChIP-PCR**	**RT-PCR**	
Mouse male pCTX	Repeated METH (1 mg/kg, s.c.) for 7 d, co-treatment with modafinil (90 mg/kg i.p.)	↑ METH vs. CT			*Drd1, Hctr1, and Hrh1*	*Drd1, Gria1, and Grin1*	[64]
		↓ METH vs. CT	pan-H3ac and pan-H4ac	*Drd2, Hctr1, Hctr2, Hrh1, Hrh3, and Grin1*		*Hctr2*	
		↑ METH + Mod vs. CT		*Adra1a*	*Drd1, Hctr1, and Hrh1*		
		↓ METH + Mod vs. CT		*Hrh3*		*Hctr2*	
			**WB**	**pan-H3ac Enrichment ChIP-PCR**	**pan-H4ac Enrichment ChIP-PCR**	**RT-PCR**	
Mouse male pCTX	METH (5 mg/kg × 1-development phase) for 7 d, withdrawal for 7 d followed by challenge METH (1 mg/kg x1), s.c.	↑ METH vs. CT	ANP32A and POUF3F2	POUF3F2 (*challenge phase*)	ANP32A (*all three phases*) and POUF3F2 (*challenge phase*)	*NP32A and POUF3F2 (development phase)*	[65]
			**WB**	**ChIP-PCR**	**RT-PCR**		
Rat male DStr	METH escalated doses (0.5 mg/kg to 3 mg/kg) for 3 wk, co-treatment with sodium valporate (VPA-300 mg/kg) followed by challenge METH (5 mg/kg), 1.p.	↑ METH vs. CT	HDAC1, HDAC2, SIRT1, SIRT2, CoREST and REST	*HDAC1* enrichment on *GluN1* *HDAC2* enrichment on *GluA1* and *GluA2* *CoREST* enrichment on *GluA1* and *GluA2* *REST* enrichment on *GluN1*			[66]
		↓ METH vs. CT	GluA1, GluA2, GluN1, H4K5ac, H4K12ac, and H4K16ac	*H4K5ac* enrichment on *GluA2* and *GluN1* *H4K12ac* enrichment on *GluA1, GluA2*, and *GluN1 H4K16ac* enrichment on *GluA1, GluA2,* and *GluN1*	*GluA1*, *GluA2*, and *GluN1*		
		↑ METH + VPA vs. METH		*H4K16ac* enrichment on *GluA1*	*GluA2* and *GluN1*		
			**RT-PCR**				
Rat male DStr	METH (10 mg/kg × 4 every 2 h) i.p.	↓ METH vs. CT	*Hdac6, Hdac8, Hdac9, Hdac10, Sirt2, Sirt5, Sirt6, and Hdac11*				[67]
			**RT-PCR**				
Rat male DStr and NAc	METH SA (0.1 mg/kg/infusion, 9 h/d) for 20 d followed by footshock (5 d) and withdrawal for 30 d	↑ SR vs. CT	*DStr—Kat4/Taf1, Kat5/Tip60*				[69]
	SR-Footshock resistant/compulsive METH taking rats	↓ SR vs. CT	*DStr—Hdac5, Hdac7, and Hdac10* *NAc—Hdac1 and Hdac11*				
	SS-Footshock sensitive/abstinent rats	↑ SR vs. SS	*DStr—Kat4/Taf1, Kat5/Tip60, Hdac2, Hdac8 and Hdac9*				
			**RT-PCR**				
Rat male DStr	METH SA (0.1 mg/kg/infusion, 9 h/d) for 10 d and withdrawal for 30 d. Seven days prior to SA, AAV injection for overexpression HDAC5 (mutant/mHDAC5) or knock-down HDAC5 (short-hairpin/shHDAC5)	↑ shHDAC5 vs. CT	*Hdac1, Hdac4, Gnb4, and Suv39h1*				[74]
		↓ shHDAC5 vs. CT	*Hdac5 and Tacr1*				
			**WB**	**pCREB enrichment ChIP-PCR**			
Rat male DStr	METH SA (0.1 mg/kg/infusion, 15 h/d) for 8 d) and withdrawal for 2 h, 24 h, and 30 d	↑ METH vs. CT	2 h and 24 h—H3K4me3 and pCREB	*2h- c-Fos, FosB, Bdnf*, and *Syp*			[79]
		↓ METH vs. CT	30d- pCREB				
			**H3K4me3 enrichment ChIP-PCR**	**RT-PCR**			
Mouse male NAc	METH (2 mg/kg, once every 96 h) for 5 times, s.c. and withdrawal for 7 wk	↑ METH vs. CT	Ccr2	*Ccr2*			[83]
			**WB**	**H3K4me3 enrichment ChIP-PCR**	**Microarray**		
Mouse male NAc	METH (2 mg/kg i.p., 2 CPP training session/day) for 3 d.	↑ METH vs. CT	H3K4me2, H3K4me3, pan-H2ac, and pan-H3ac	*Oxtr*	*Mll1/Kmt2a, Oxtr and c-Fos*		[87]
	Prior to METH CPP training intra-NAc infusion of Kdm5c siRNA for knock-down (Kdm5c KD)	↑ METH Kdm5c KD vs. CT	H3K4me3				
	Prior to METH CPP training intra-NAc infusion of Mll1/Kmt2a siRNA for knock-down (Mll1 KD)	↓ METH Mll1/Kmt2a KD vs. CT	H3K4me3				
			**HDAC enzymatic assay**				
Rat female mAmygdala	METH injection (5 mg/kg × 1) for 4 d i.p., co-treatment with estradiol benzoate (EB) and progesterone (P) for 5 h s.c.	↓ METH + EB + P vs. CT	HDAC activity				[89]
			**pan-H3ac enrichment ChIP-PCR**	**pan-H4ac enrichment ChIP-PCR**	**RT-PCR**		
Mouse male pCTX	Single dose METH (1 mg/kg) for 1h, Repeated METH (1 mg/kg × 1) for 7 d s.c.and withdrawal for 4 d, co-treatment with modafinil (90 mg/kg) i.p.	↑ single dose METH vs. CT		*Hdac1 and Hdac10*	*Hdac1, Hdac2, Hdac4, Hdac5, and Hdac8*		[90]
		↓ single dose METH vs. CT		*Hdac2, Hdac4, Hdac5*, and *Hdac8*			
		↑ single dose METH + Mod vs. CT	*Hdac7*		*Hdac1, Hdac2, Hdac5, Hdac7 and Hdac8*		
		↑ rep. dose METH vs. CT	*Hdac3, Hdac4, Sirt3, and Sirt6*	*Hdac1, Hdac4, Hdac5, and Hdac11*	*Hdac4 and Sirt7*		
		↓ rep. dose METH vs. CT	*Hdac1, Hdac2, and Hdac8*	*Hdac2 Hdac8, and Sirt7*	*Hdac2*		
		↑ rep.dose METH + Mod vs. CT	*Hdac3, Hdac4, and Sirt6*		*Hdac1, Hdac2, Hdac5, Hdac7 and Hdac8*		
		↓ rep. dose METH + Mod vs. CT		*Hdac8*			
			**RNA-Seq**				
Rat male CeA	METH SA (0.1 mg/kg/infusion, 9 h/d) for 10 d, and withdrawal for 35 d.	↑ METH vs. CT	*Mll1/Kmt2a*				[91]

Abbreviation: METH, methamphetamine; CeA, central amydala; DStr, dorsal striatum; mAmygdala, medial amygdala; pCTX, medial prefrontal cortex; NAc, nucleus accumbens; i.p., intraperitoneal; s.c., subcutaneous; ↑, significantly increased; ↓, significantly decreased; CT, control; WT, wild-type; KD, Knock-down; KO, knock-out; AAV, adeno-associated virus; SA, self-administration; h, hour(s); d, day(s); wk, week(s); WB, Western Blot/immunoblot; HAT, histone acetyltransferases; HDAC, histone deacetylases; ChIP-PCR, chromatin immunoprecipitation PCR; ChIP -Seq, chromatin immunoprecipitation sequencing; RT-PCR, reverse transcriptase-polymerase chain reaction.

### 2.3. DNA Methylation

In 1948, Rollin Hotchkiss first detected chemical modifications on the fifth position of cytosine DNA base, where hydrogen group was replaced by methyl group, and suggested that this modified methyl cytosine existed naturally in DNA [92]. Involvement of DNA methylation in gene regulation was not explored until the 1980s when it came into the limelight with studies that demonstrated its role in gene regulation and cell differentiation [93,94]. This reversible process of DNA methylation is catalyzed by DNA methyltransferase (DNMT) [94]. The added methyl group does not affect the base pairing itself, but the protrusion of methyl groups into the DNA major groove can affect DNA–protein interactions [95].

#### 2.3.1. Pre-Clinical Studies

Animal models studying the effects of METH on DNA methylation (Table 2) revealed that acute and chronic METH injections increased striatal Dnmt1 mRNA expression in a strain-specific manner (increased in the Fisher 344 and not in the Lewis strain) [96]. Similarly, Jayanthi et al. (2018) [51] validate this finding of increased striatal Dnmt1 mRNA in their study using single dose of METH in Sprague Dawley rats [52].

Enzyme-linked immunosorbent assay (ELISA—based DNA methylation determination found increased 5-mC levels in the pCTX after chronic METH exposure [64]. A recent study using a pyrosequencing method to measure DNA methylation marks in chronic METH in pCTX and hippocampus found that several CpG sites in the IEGs including Arc, Fos, Klf10, and Nr4a1 had significant changes in their DNA methylation levels [97]. Itzhak et al. (2015) [98] found the extended role of hippocampal DNA methylation by studying behavioral response after in-utero METH exposure and identified several candidate genes involved in cognition and memory alteration in DNA methylome [98].

Using methylated DNA immunoprecipitation (MeDIP), Jayanthi et al. (2014; 2018 and 2020) [52,66,99] had shown changes in DNA methylation after different paradigms of METH. Acute injection of METH followed by a 30-day withdrawal period led to increased mRNA expression of the stress-related genes Crh and Avp due to DNA hypomethylation at CpG sites near the promoter region for Crh and at intragenic region for Avp [52]. In association with DNA methylation, chronic METH increased methylated CpG binding protein 2 (MeCP2) protein levels and caused MeCP2 co-precipitation with HDAC2. The MeCP2- HDAC2 protein led to transcription repression of AMPA glutamate receptors (GluA1 and GluA2). This observation was supported by ChIP assay that showed METH increased MECP2 enrichment at the promoter regions of GluA1 and GluA2. Moreover, MeDIP PCR revealed decreased cytosine methylation at CpG sites located near the promoter site of GluA2 and at CpG-rich site located at −23 kb from promoter region for GluA1 [66].

To model the escalation of drug-use criterion of MUD, Jayanthi et al. (2020) [99] used noncontingent injections of METH before SA training and found that METH pre-treatment caused enhancement of escalated METH SA and down-regulated mRNA and protein expression of voltage-gated K+ channels (Kcna1, Kcna3, and Kcnn1). The epigenetic mechanisms underlying the transcriptional alterations observed may be due to increased DNA methylation at the CpG-rich sites on their promoter sequences [99]. In addition to changes in the expression of stress-related genes- Crh, Avp, AMPA glutamate receptors and voltage-gated K+ −channels recently, the hallmark protein in Parkinson’s disease SCNA was significantly increased after exposure to high, prolonged dose of METH. The elevation in gene expression was associated with hypomethylation within the SCNA promoter sequences [100].

Beyond changes in CpG sites, the long-interspersed element-1 (LINE-1) in the DNA known to cause genome instability when altered was found to be regulated by DNA methylation and histone modifications. According to the report by Moszczynska et al. (2015) [101], METH injections exhibit increased activity of LINEs in the dentate gyrus of hippocampus and DStr regions that contribute to METH-induced impairment in cognition and memory [101].

In addition, reports from Mong’s lab demonstrated that repeated exposure to METH enhances sexual motivation in hormonally primed female rats on an epigenetic level [88]. They observed a significant increase in DNMT3b protein level in female rats treated with METH and exogenous steroids in comparison to METH-alone treated rats [89]. This suggests that increased sex drive is not a consequence of METH use but could be the reason behind drug use.

#### 2.3.2. Clinical Studies

Detection of DNA methylation levels by methylight qPCR in male METH addicts revealed significant correlation between the methylation levels of chimerin 2 (CHN2) and METH dependence [102]. CHN2 is a protein involved in remodeling of the actin cytoskeleton and hippocampal axonal pruning [103]. This suggests that prolonged abuse of METH induces abnormal methylation of CHN2 gene that interferes in actin skeleton remodeling leading to irregular formation of neurites and growth cones that is considered crucial to maintaining long-lasting addictive behavior [104].

Another study that explored the genome-wide methylation analysis of METH users differentiated by their clinical diagnostic criteria qualification for MUD. Subjects that do meet the criteria belong to low METH addictive quality group (LMAQ) and the ones who qualified for METH dependence as high METH addictive quality group (HMAQ). The two addiction phenotypes (LMAQ and HMAQ) was distinctly segregated by the percentage of methylated Caveolin-2 (CAV2) that was significantly increased only in the low addictive group [105].

Pyrosequencing studies showed a significant increase in parvalbumin (PVALB) methylation levels in the psychotic subgroup of METH-dependent patients and no change was seen in the non-psychotic METH group. The change in PVALB level might contribute to the GABAergic deficits associated with METH dependence [106]. In addition, Nohesara et al. (2016) [107] found promoter DNA hypomethylation of candidate genes involved in DA regulation (DRD3, DRD4, MB-COMT, and AKT1) associated with increased expression of the corresponding genes in psychotic METH-dependent patients [107]. Another molecular mediator of memory consolidation process linked to psychostimulants that was found hypomethylated in METH addicts was the brain-derived neurotrophic factor (BDNF) [108]. Among the five CpGs (CpG1–5) measured on the BDNF promoter using pyrosequencing analysis only CpG5 methylation was significantly reduced in METH abusers. Moreover, the report also revealed a functional connection between the methylation of the CpG5 fragment and Bdnf gene expression level using cell-based luciferase assay [108]. Interestingly, a research group from Japan [109] has recently identified the gene Shati/Nat8L as a medical marker for MUD diagnosis after gaining support from several pre-clinical research data [110,111,112]. Research by Yuka et al. (2020) [109] reported that the ratio of DNA methylation in SHATI/NAT8L was significantly higher at six CpG sites in METH users when compared to healthy subjects [109].

One of the significant METH-related public health consequences concerns the long-term effects of METH on brain development and associated behaviors in children born to addicted mothers. This issue was addressed by a longitudinal study in children with in-utero exposure to METH. The authors used sodium bisulfite pyrosequencing and found increased DNA methylation at the CpG2 site of HSD11B2, a stress-related gene [113].

Another study has reported that METH-induced changes in LINE-1 methylation patterns were associated with METH-induced paranoia [114]. Thus, it is possible that METH-induced neuro-oxidative pathways may have altered LINE-1 partial methylation patterns, which in turn may increase risk to develop METH-induced paranoia [114] (see Table 2 for details).

### 2.4. DNA Hydroxymethylation

DNA hydroxymethylation (5-hmC), was discovered in T-even bacteriophage [115] and later in rats in 1972 [116]. Only recently 5-hmC has been implicated in gene regulation and has been identified as a novel epigenetic mark [117]. In 5-hmC the methyl group of cytosine is replaced by a hydroxy-methyl group catalyzed by the ten-eleven-translocation (TET) family of proteins [118]. Significant levels of 5-hmC, a ten-fold enrichment was observed in the brain [119,120,121] especially within gene bodies that are strongly transcribed [122,123].

Several studies have focused on the role of 5-hmC in learning and memory using conditional knockout of the Tet genes [124,125,126,127]. Though understudied, Cadet and his team have published data on METH-mediated effects on 5-hmC [52,66,128]. Using a rat model of METH SA and footshocks as adverse consequences, they found that METH-addicted rats showed differential DNA hydroxymethylation in the NAc in comparison to both control and METH-abstinent rats (rats that suppressed METH intake after footshock). These changes occurred mostly at intergenic sites located on long interspersed elements (LINEs). Moreover, they also observed differential DNA hydroxymethylation and increased expression of specific members of potassium channels in the NAc that appear to be promoters of the abstinence from METH-taking behaviors [128].

Using experimenter-administered doses of METH, the same group documented increased DNA hydroxymethylation at the promoter region of the stress gene, corticotrophin-releasing hormone (Crh), and at the intragenic DNA sequence of vasopressin (Avp) [52]. Interestingly, there were also METH-induced increased TET1 and TET3 levels in the NAc. Importantly, METH increased TET1 binding at the Crh promoter and increased TET3 binding at Avp intragenic regions suggesting that TET-induced DNA hydroxymethylation is an important driver of the effects of METH in the NAc. The use of the TET inhibitor, 1,5-isoquinolinediol (IQD) provided conclusive evidence that the TETs were involved in the regulation of Crh and Avp mRNA expression levels [52]. In contrast, increasing doses of METH over a period of two weeks led to decreased enrichment of 5-hmC at the promoter regions of striatal AMPA glutamate receptors in rats [66].

**Table 2:** DNA modifications affected by METH exposure.

Source	Experimental Design	Comparison	Assay and Results				Ref.
			**WB**	**ChIP-PCR**	**MeDIP and hMeDIP-PCR**	**RT-PCR**	
Rat male NAc	Single dose METH (10 mg/kg) for 1, 2, 3, and 4 wk, co-treatment with 1,5-isoquinolinediol (IQD, 3 mg/kg) on days 1, 2, 4, and 6 per wk, i.p.	↑ METH vs. CT	TET1 DNMT1 (2–4 wk) TET2 and TET3 (4 wk)	TET1 enrichment on Crh TET3 enrichment on Avp	hMeDIP- Crh and Avp (4 wk)	*Cartpt and Crh* (2–4 wk) *Avp* (3–4wk)	[52]
		↓ METH vs. CT			MeDIP- Crh and Avp (4 wk)		
		↑ METH + IQD vs. CT				*Avp and Crh* (4 wk)	
			**ELISA**				
Mouse pCTX	Repeated METH (1 mg/kg, s.c.) for 7 d, co-treatment with modafinil (90 mg/kg i.p.)	↑ METH vs. CT	Global 5mC				[64]
			**WB**	**ChIP-PCR**	**MeDIP and hMeDIP-PCR**		
Rat male DStr	METH escalated doses (0.5 mg/kg to 3 mg/kg) for 3 wk, co-treatment with sodium valporate (VPA-300 mg/kg) followed by challenge METH (5 mg/kg), 1.p.	↑ METH vs. CT	MeCP2, and DNMT1	MeCP2 enrichment on GluA1 and CoREST enrichment on GluA1 and GluA2			[66]
		↓ METH vs. CT			MeDIP- GluA2 hMeDIP- GluA1 and GluA2		
			**WB**				
Rat female VMN	METH injection (5 mg/kg × 1) for 4 d i.p., co-treatment with estradiol benzoate (EB) and progesterone (P) for 5 h s.c.	↑ METH + EB + P vs. METH	DNMT3b				[89]
		↑ METH + EB + P vs. CT + EB + P	DNMT3b				
			**Pyrosequencing**	**PCR Array**	**RT-PCR**		
Mouse male pCTX and HIP	Repeated METH (2 mg/kg x 16) injected every other day for 31 d, i.p. and withdrawal for 1 wk. DNA methylation levels on CpG islands via pyrosequencing.	↑ METH vs. CT	pCTX—Arc (site # 3, 5, and 9) and c-FosHIP—Klf10				[97]
		↓ METH vs. CT	pCTX—Arc (site # 4, 6, 7, and 10) HIP—Nr4a1	pCTX—*Arc, Bdnf, Cebpb, Egr1, 2, and 4, c-Fos, Junb, Klf10, Ngf, Nr4a1,and Pim1* HIP—*Arc, Egr2, and 3, c-Fos, c-Jun, Klf10, Nr4a1,and Plat*	pCTX and HIP—*Arc, Egr2, c-Fos, and Nr4a1*		
			**Pyrosequencing**				
Mouse male and female, HIP	METH escalated doses (0.5 to 4 mg/kg, i.p.) from PD33 to PD59 followed by mating on PD60 and from PD61 injected with fixed dose of METH (4 mg/kg, s.c.) every-other-day for 17 d.	↑ MpMd vs. SpSd	Bcl7c, Dhx16, Hspb8, Pgam1, Six6, Snx7, and Txnrd3				[98]
	*SpSd*-Saline pups reared by saline exposed dams*; MpMd-* METH pups reared by METH exposed dams	↓ MpMd vs. SpSd	Col24a1 and Hdac5				
			**WB**	**MeDIP-PCR**	**RT-PCR**		
Rat male Nac	Single dose METH (10 mg/kg, i.p.) followed by METH SA (0.1 mg/kg/infusion) for 18 d and withdrawal for 30 d	↑ MM vs. SM		*Kcna1, Kcna3 and Kcnn1*	*Kcna4, Kcna5, Kcnn3, Kcnma1, and Kcnmb2*		[99]
	SM- Single saline injection followed by METH SA	↓ MM vs. SM	*Kcna1, Kcna3, and Kcnn1*		*Kcna1, Kcna3, Kcna5, Kcna6, and Kcnn1*		
	MM- Single METH injection followed by METH SA	↑ SM vs. CT	*Kcna1, Kcna3, and Kcnn1*		*Kcna1, Kcna3, Kcna5, Kcna6, Kcnb1, Kcnb2, Kcnn1, Kcnn3, Kcnma11, and Kcnmb2*		
		↓ SM vs. CT		Kcna1			
			**WB**	**IHC**	**TEM**	**MeDIP-PCR**	
Mouse male striatal neurons	Acute METH (5 mg/kg × 5 every 2 h) and withdrawal for 1 h, 2 d, and 7 d Sub-acute METH (5 mg/kg × 1 daily) for 7 d and withdrawal for 1 d, 7 d, and 21 d Chronic METH (5 mg/kg × 1 daily) for 21 d and withdrawal for 1 d, 7 d, and 21 d	↑ METH vs. CT	α- SYNUCLEIN Acute—1 h Sub-acute—7 d and 21 d Chronic—1 d, 7 d, and 21 d	α- SYNUCLEIN Acute—1 h Sub-acute—7 d and 21 d Chronic—1 d, 7 d, and 21 d	α- SYNUCLEINAcute—1 h Chronic—1 d, 7 d, and 21 d		[100]
		↓ METH vs. CT	α- SYNUCLEIN Acute—7 d	α- SYNUCLEIN Acute—1 h and 7 d		5-mC enrichment on SCNA Acute—1 h Sub-acute and Chronic—1 d, 7 d, and 21 d	
			**Quantitative methylation specific PCR**	**Quantitative non-methylation specific PCR**			
Male human peripheral blood	METH abusers and healthy controls.	↑ METH vs. CT	CHN2				[102]
		↓ METH vs. CT		CHN2			
			**MethyLight qPCR**				
Male human peripheral blood	METH abusers and healthy controls.	↑ LMAQ vs. CT	CAV2, LNX1, and BHLHB9				[105]
	As per DSM IV METH abusers were segregated to: Low METH addictive quality group (LMAQ); High METH addictive quality group (HMAQ)	↓ LMAQ vs. CT	SLC1A6 and PCSK9				
		↑ HMAQ vs. CT	LNX1 and BHLHB9				
		↓ HMAQ vs. CT	SLC1A6 and PCSK9				
			**PyroMark PCR**				
Male human blood cells	METH abusers and healthy controls.	↑ Psy vs. CT	PVALB (CpG1 and CpG2)				[106]
	As per DSM IV METH abusers were segregated to: with psychosis (Psy) and without psychosis (Non-Psy)						
			**MeDIP-PCR**	**RT-PCR**			
Male and female human saliva	METH abusers and healthy controls.	↓ Psy vs. CT	*DRD3, DRD4, MB-COMT, and AKT1*				[107]
	As per DSM IV METH abusers were segregated to: with psychosis (Psy) and without psychosis (Non-Psy)	↓ Non-Psy vs. CT	*AKT1*				
		↑ Non-Psy vs. Psy	*DRD3, DRD4, MB-COMT, and AKT1*				
		↑ Psy vs. CT		*DRD2, DRD3, DRD4,MB-COMT, and AKT1*			
		↑ Non-Psy vs. CT		*MB-COMT and AKT1*			
		↓ Non-Psy vs. Psy		*DRD4*			
			**Pyrosequencing**				
Male and female human peripheral blood	METH abusers and healthy controls	↑ METH vs. CT	Methylation of CpG of BDNF in site # 1–4				[108]
			**Pyrosequencing and PyroMark PCR**				
Male and female human peripheral blood	METH abusers with psychotic disorder and healthy controls	↑ METH vs. CT	Methylation of CpG of *SHATI/NAT8L* in site # 2, 4–8				[109]
			**PyroMark PCR**				
Male and female human saliva	10 and 11-year old children, with prenatal METH exposure (PME) and healthy controls	↑ PME vs. CT	Methylation of CpG2 of *HSD11B2* in model 1 and 2. The effect observed remained after adjusting for cortisone and co-variates				[113]
	The PME children were separated into 4 model groups: Model 1—Only PMEModel 2—PME + Early adversity Model 3—PME + Early adversity + Cortisone Model 4—PME + Early adversity + Cortisone + Co-variates (gestational age, cigarette, alcohol and marijuana use)						
			**COBRA on LINE-1**				
Male and female human peripheral blood	METH abusers and healthy controls.	↓ Non-Psy vs. CT	% uCmC				[114]
	As per DSM IV METH abusers were segregated to: with psychosis (Psy) and without psychosis (Non-Psy)	↓ Psy vs. Non-Psy	% uCmC				
	LINE-1 alleles are classified into four patterns: hypermethylation (mCmC) partial methylation 5′m with 3′u (mCuC) partial methylation 5′u with 3′m (uCmC) hypomethylation (uCuC)	↑ Non-Psy vs. CT	% mCuC % mCuC + % uCmC				
			**hMeDIP-Seq**	**RT-PCR**			
Rat male Nac	METH SA (0.1 mg/kg/infusion, 9 h/d) for 20 d followed by footshock (10 d)	↑ SR vs. CT	*Kcnb2 and Kcnn2*				[128]
	SR-Footshock resistant/compulsive METH taking rats	↑ SS vs. CT	*Kcnip2, Kcnj2, Kcnj3, Kcnk12, Kcnma1, and Kcnn2*	*Kcna1, Kcna2, Kcnb2, Kcnma1, Kcnn1, and Kcnn2*			
	SS-Footshock sensitive/abstinent rats	↑ SR vs. SS	*Kcna4, Kcnb2, Kcnd3, Kcnh1, Kcnk1, Kcnn2, and Kctd13*				
		↓ SR vs. SS	*Kcnb2, Kcnn2, and Kcnt2*	*Knca1, Kcna2, Kcnab1, Kcnb2, Kcnma1, Kcnn1, and Kcnn2*			

Abbreviation: METH, methamphetamine; DStr, dorsal striatum; pCTX, frontal cortex; HIP, hippocampus; NAc, nucleus accumbens; VMN, ventromedial nucleus of the hypothalamus; i.p., intraperitoneal; s.c., subcutaneous; ↑, significantly increased; ↓, significantly decreased; CT, control; SA, self-administration; h, hour(s); d, day(s); wk, week(s); DNMT, DNA methyltransferases; CHN2, chimeric protein 2; 5mC, 5-methylcytosine; RT-PCR, reverse transcriptase PCR; MeDIP-PCR, methylcytosine DNA immunoprecipitation PCR; hMeDIP-PCR, hydroxylmethylcytosine DNA immunoprecipitation PCR; hmC-Seq, hydroxylmethylcytosine DNA immunoprecipitation sequencing; ChIP-PCR, Chromatin immunoprecipitation PCR; WB, Western Blot (immunoblot); IHC, immunohistochemistry; COBRA, combined bisulfite restriction analysis; LINE-1, long interspersed element-1.

### 2.5. Non-Coding RNA

Potential involvement of non-coding RNA (ncRNA) have not yet been fully examined in MUD. Elucidation of their potential roles may also impact therapeutic options for MUD.

Non-coding RNAs refer to RNAs that do not translate into proteins but still perform crucial roles in transcription and post-transcriptional events [129]. These include microRNAs (miRNAs), small interfering RNAs (siRNAs), small nuclear RNAs (snRNAs), nucleolar RNAs (snoRNAs), and long non-coding RNA (lncRNA) [130]. The accumulated evidence supports the role of miRNAs (15–25-nucleotides in length) as regulators of genes involved in METH-mediated changes in dendritic spines, synaptic transmission [131,132], and unfolded protein response [133] (see Table 3 for details).

#### 2.5.1. Pre-Clinical Studies

A preclinical study using conditioned place preference (CPP) has reported that METH CPP is accompanied by the upregulation of 276 and downregulation of 25 miRNAs in serum exosomes [134]. Using the KEGG pathway analysis, the authors found that these miRNAs-regulated genes are involved in vesicular transport, amphetamine addiction, cGMP-PKG signaling pathway, dopaminergic synapse, and GABAergic synapse [134]. Another study by Qian et al. (2021) [132] found that chronic administration of METH to mice induced miR-31-3p in the dorsal HIP. This miRNA targets RhoA, an enzyme that mediates actomyosin signaling, vesicular trafficking, and dendritic spine morphology. Interestingly, overexpression of miR-31-3p increased METH-induced CPP score whereas miR-31-3p knockdown attenuated CPP in mice [132]. Using the CPP model in rats, Yang et al. (2020) [135] also measured the expression of a large number of miRNAs in the NAc and showed changes in miRNAs that target genes involved in Wnt signaling, tuberculosis, toxoplasmosis, spliceosome, and axon guidance [135]. Moreover, Wang et al. (2021) [133] showed that METH-CPP was associated with the downregulation of miRNA, miR-181a-5p, in the DStr. They also identified 36 target genes from online bio-informatic databases and were able to validate up-regulation of 11 of these genes that are members of the ER chaperone complex [133]. These results support other experiments that have demonstrated that METH can impact the function of the ER.

It is of interest that miRNA expression profiling in the DStr of METH-injected rats using high-throughput sequencing analysis identified 167 miRNAs that were dysregulated (113 up-regulated and 54 down-regulated). Using network enrichment analysis, the authors reported changes in the expression of genes that regulate PI3K-Akt and FoxO signaling [136]. In addition to changes in gene expression in the NAc and DStr, miRNA profiling is also altered in the midbrain that mainly contributes to dopaminergic signaling in MUD [137]. Using short access of drug self-administration over a period of 40 days, Bosch et al. (2015) [137] reported that METH SA was accompanied by 7 up-regulated and 71 down-regulated miRNAs in the VTA. Most of these genes participated in dopamine metabolic process, biological quality, and plasma membrane integrity [137].

METH intake also dysregulates miRNA biogenesis. Specifically, METH-induced behavioral sensitization in mice is associated with decreased Argonaute2 (Ago2) mRNA expression [138]. These changes were negatively correlated with the development phase of behavioral sensitization [138]. Measurements of Ago2-dependent miRNAs in NAc neurons found that miR-3068-5p could disrupt METH-induced locomotor sensitization. These effects of Ago2/miR-3068-5p occurred through interactions with the glutamate receptor, GluN1/Grin1 [138]. Li et al. (2020) [131] also found increased expression of miR-128 during METH-induced locomotor sensitization. Moreover, AAV-mediated overexpression of miRNA-128 had significant additional effects on the locomotor activity [131]. In contrast, AAV-mediated inhibition of miRNA-128 attenuated METH-induced locomotion. Interestingly, the researchers identified three differentially expressed proteins (Arf6, Cpeb3, and Nlgn1) in miR-128-dependent METH-induced sensitization. Importantly, these proteins are involved in controlling dendritic morphology and synaptic transmission [131] that are known to be impacted by METH administration.

It is important to note that a study has suggested that METH might impact the expression of miRNAs in extracellular vesicles (EV) [139]. METH-dependent CPP in mice caused an increased expression of EV-containing miRNAs (miR-183-5p, miR-9a-5p, and miR-369-3p) in the hippocampus [140]. These miRNAs are known to play a crucial role in cell communication in the CNS and peripheral system [140]. These investigators also showed decreased hippocampal protein levels of ErbB4 and NRG1, that might serve as markers of METH-induced psychosis [141,142].

#### 2.5.2. Clinical Study

Studies in humans with MUD have documented changes in plasma EVs. Specifically, Sandau et al. (2020) [143], using a miRNA array platform, reported differential expression (19 up-regulated and 69 down-regulated) of miRNAs in the peripheral blood of female patients. The authors found that age of first use was correlated positively with changes in miR-628-5p expression but negatively correlated with miR-301a-3p and miR-382-5p. In addition, lifetime of METH use correlated positively with miR-301a-3p and miR-382-5p expression but negatively with miR-628-5p expression. Finally, the frequency of METH use was negatively correlated with miR-382-5p [143]. This study illustrates the need to collect all essential data regarding the clinical history of patients who meet the criteria for a substance-use disorder diagnosis.

A recent study by Chen et al. (2021) [144] using plasma EVs of METH patients reported potential relationships between symptoms of anxiety and depression and changes in miRNAs in EVs. Specifically, changes in anxiety and depression scores were negatively correlated with the expression of miR-363-3p, miR-16-5p, miR-129-5p, and miR-92a-3p [144]. These studies support, in part, the idea of using changes in EVs as potential biomarkers of psychiatric disorders, mainly anxiety and depression, in patients exposed to METH [143,144]. Before such conclusions can be reached, however, there is a need to carry out large-scale clinical studies that compare METH-associated anxiety and depression to other clinical populations that have not been exposed to any psychostimulant. Another study conducted by Gu et al. (2020) [145] compared serum miRNA expression profiles in male and female METH-dependent patients to those of normal controls. They reported 5 up-regulated and 9 down-regulated miRNAs. More clinical work is necessary to identify specific mRNAs and non-coding RNAs whose expression might be altered in plasma EVs. These studies promise to provide potential windows to occurrences in the CNS. Such panoramic views may help to decipher the molecular substrates of MUD and help to better plan therapeutic interventions against this malady that affects so many patients.

**Table 3:** Non-coding RNAs affected by METH exposure.

Source	Experimental Design	Comparison	Assay and Results				Ref.
			**Behavioral Sensitization**	**RT-PCR**	**miRNA-Target Protein Interaction**	**WB**	
Mouse male NAc	METHiHS- Chronic METH for 5 d (2 mg/kg, once daily), i.p. or and withdrawal for 2 d, followed by METH challenge (2 mg/kg) i.p.	↑ ChMETH vs. CT	Yes	*Arf6*			[131]
		↓ ChMETH vs. CT		*Nlgn1*		Arf6 and Nlgn1	
	Saline for 7 d followed by Acute METH (2 mg/kg), i.p.	↑ ChMETH vs. AcMETH		*Cpeb3*			
		↓ ChMETH vs. AcMETH		*Arf6*			
		↑ AcMETH vs. CT		*Arf6*			
		↓ AcMETH vs. CT		*Cpeb3*			
	AAV-miR-128 KD	↑ KD CT vs. CT				Nlgn1	
		↑ KD METH vs. ChMETH			100	Arf6, Nlgn1, and Cpeb3	
		↓ KD METH vs. ChMETH	Yes		18		
	AAV-miR-128 KI	↓ KI CT vs. CT				Nlgn1	
		↑ KI METH vs. ChMETH	Yes				
		↓ KI METH vs. ChMETH				Arf6 and Cpeb3	
			**WB**	**miRNA-PCR**	**CPP Score**		
Mouse male dHIP	METH CPP for 8 d (1 mg/kg, every other day), i.p.	↑ METH vs. CT		*miR-31-3p*	Post-test		[132]
		↓ METH vs. CT	RhoA				
	AAV-RhoA- or AAV-miR-31-3p- KD	↑ KD CT vs. CT	RhoA (miR-31-3p)				
		↓ KD METH vs. KD CT	RhoA (miR-31-3p)				
		↑ KD METH vs. METH	RhoA (miR-31-3p)		Post-test (RhoA)		
		↓ KD METH vs. METH			Post-test (miR-31-3p)		
	AAV-RhoA- or AAV-mIR-31-3p- KI	↓ KI CT vs. CT	RhoA (miR-31-3p)				
		↑ KI METH vs. METH			Post-test (miR-31-3p)		
		↓ KI METH vs. METH	RhoA (miR-31-3p)		Post-test (RhoA)		
			**miRNA-PCR**	**RT-PCR**			
Rat male DStr	METH CPP for 8 d (1 mg/kg, every other day), i.p.	↑ METH vs. CT		*Ube2d3, Rnf169, Fbxo33, Rad23b, Neurl1b, Pcnp, Tulp4, Klhl15, Rnf34, Derl1, and Hsp90b1*			[133]
		↓ METH vs. CT	rno-miR-: 181a-5p and 181b-5p				
			**miRNA-Seq**	**miRNA-PCR**			
Mouse NAc	METH CPP for 4 d (2 mg/kg), s.c.	↑ METH vs. CT	276	miR-: 197-5p, 22-3p, 152-3p and 218b			[134]
		↓ METH vs. CT	25				
			**miRNA-Seq**	**miRNA-PCR**	**RT-PCR**		
Rat male NAc	METH CPP for 4 wk (10 mg/kg, twice daily), i.p.	↑ METH vs. CT	17 miRs	rno-miR-: 217-5p, 31b, 28-3p, 31a-5p, 547-3p, and 216b-5p	*5-Htr1b, Rtn4, and Sv2a*		[135]
		↓ METH vs. CT	23 miRs	rno-miR-: 1b, 144-5p, 202-5p, 133a-3p, 133c, and 451-5p	Rbm8a and Syt7		
			**miRNA-Seq**	**High target gene #**			
Rat male DStr	METH for 3 d (2 mg/kg), i.p. followed by METH for 4 d, (5 mg/kg), i.p.	↑ METH vs. CT	113	rno-miR-: 3068-5p, 34a-5p, 326-3p and let-7b-5p			[136]
		↓ METH vs. CT	54	rno-miR-485-5p			
			**mRNA expression Array**	**RT-PCR**	**miRNA-Array**		
Rat male VTA	METH SA for 40 d (0.1 mg/kg/infusion, 2 h/d, FR-1 to FR-5)	↑ METH vs. CT	31	*Ret and Dat*	7		[137]
		↓ METH vs. CT	17		71		
			**RT-PCR**	**miRNA-PCR**			
Mouse NAc	METHiHS -Repeated METH for 5 d (2 mg/kg), i.p. and withdrawal for 2 d, followed by METH challenge (2 mg/kg), i.p.	↓ METH vs. CT	*Ago2*				[138]
	AAV-SYN-Ago2 KI	↑ Ago2 KI vs. CT	*Ago2 and Grin1*	miR-: 3068-5p and 30a-5p			
		↓ Ago2 KI vs. CT		miR-: 124-3p, 33-5p, and 376a-3p			
	AAV-Ago2 KD	↑ Ago2 KD vs. CT		miR-: 33-5p and 376a-3p			
		↓ Ago2 KD vs. CT	*Ago2*	miR-3068-5p			
	AAV-SYN-spmIR-3068	↑ miR-3068 KD vs. CT	*Grin1*				
		↓ miR-3068 KD vs. CT	*App*				
			**miRNA-PCR**	**WB**			
Mouse male HIP	METH CPP for 6 d (2 mg/kg), s.c.	↑ METH vs. CT	miR-: 183-5p, 9a-5p, and 369-3p				[140]
(EV containing miRNAs)		↓ METH vs. CT		ErbB4 and NRG1			
			**miRNA-Array**	**Age of onset**	**Percent of lifetime**	**Frequency of use**	
Female human peripheral blood	METH dependent patients (diagnosed according to DSM IV) and healthy controls.	↑ METH vs. CT	19				[143]
(EV containing miRNAs)		↓ METH vs. CT	69				
		Positive correlation		hsa-miR-628-5p	hsa-miR-: 301a-3p and 382-5p		
		Negative correlation		hsa-miR-: 301a-3p and 382-5p	hsa-miR-628-5p	hsa-miR-382-5p	
			**miRNA-Seq**	**miRNA-PCR**	**HAM-A**	**HAM-D**	
Human peripheral blood	METH dependent patients (diagnosed according to DSM V) and healthy controls.	↑ METH vs. CT	hsa-miR-: 151a-5p, 151b, 338-3p, 744-5p, 432-5p, and 191-3p				[144]
(EV containing miRNAs)		↓ METH vs. CT	hsa-miR-: 363-3p, 629-5p, 16-5p, 484, 486-5p, 18a-3p, 1180-3p, and 548ay-5p	hsa-miR-: 143-3p, 200a-3p, 363-3p, and 125b-5p			
		Negative correlation			hsa-miR-: 363-3p, 16-5p, 129-5p, and 92a-3p	hsa-miR-: 363-3p, 16-5p, 129-5p, and 92a-3p	
			**miRNA-Array**	**miRNA-PCR**			
Male and female human peripheral blood	METH dependent patients (diagnosed according to DSM IV) and healthy controls.	↑ METH vs. CT	hsa-miR-: 550b-3p, 9-3p, 4776-3p, 4799-3p, and kshv-miR-K12-12-3p	hsa-miR-9-3p			[145]
		↓ METH vs. CT	hsa-miR-: 3656, 4258, 1469, 1471, 4419a, 4651, 5196-5p, Plus-C1076, and ks1v-miR-H8-3p				

Abbreviation: METH, methamphetamine; DStr, dorsal STR; HIP, hippocampus; dHIP, dorsal HIP; VTA, ventral tegmental area; NAc, nucleus accumbens; i.p., intraperitoneal; s.c., subcutaneous; ↑, significantly increased; ↓, significantly decreased; CT, control; KI CT, knock-in with control vector; KD CT, knock-down with control vector; AcMETH, acute METH; ChMETH, chronic METH; KI METH, knock-in with METH treatment; KD METH, knock-down with METH treatment; EV, extracellular vesicles; AAV, Adeno-associated virus; AAV-SYN, AAV using synapsin-1 promoter for expression; AAV-SYN-spmIR-30a-5p, AAV-SYN miRNA sponge to inhibit miR-30a-5p; Rhy, rhynchophylline extract from Uncaria rhyncophylla (Miq.) Miq. ex Havil.; HAM-A, scale that grades the severity of anxiety; HAM-D, scale that grades the severity of depression; SA, self-administration; CPP, conditioned place preference; METHiHS, METH-induced hyperlocomotor sensitization; h, hour(s); d, day(s); wk, week(s); miR/miRNA, microRNA; rno, Rattus norvegicus; hsa, Homo sapiens; WB, Western Blot/immunoblot; RNA -Seq, RNA sequencing; miRNA -Seq, miRNA sequencing; RT-PCR, reverse transcriptase-polymerase chain reaction; miRNA-PCR, miRNA-polymerase chain reaction.

## 3. Conclusions

Research studies investigating the molecular effects of METH have used high-throughput sequencing to measure global epigenetic changes and altered gene expression. These studies have identified important correlations between epigenetic modifications and transcriptional changes. In most cases, combinatorial epigenetic events will be responsible for METH-induced changes in transcription in brain regions implicated in reward circuitries. Nevertheless, much more work is necessary in order to identify general and specific targets that would provide panoramic details for the various molecular switches that could trigger acceleration from recreational intake to compulsive and pathological drug use. Here, our review has summarized the accumulated evidence that histone and DNA modifications as well as changes in ncRNAs are involved in the acute and chronic effects of METH on the brain and periphery. These observations form the initial steps toward elucidating the molecular and biochemical substrates of MUD. These findings should be taken into considerations in all discussions regarding the potential development of epigenetic pharmacological agents against substance-use disorders.
